# Impact of Exposure to Chronic Light–Dark Phase Shifting Circadian Rhythm Disruption on Muscle Proteome in Periparturient Dairy Cows

**DOI:** 10.3390/proteomes9030035

**Published:** 2021-07-29

**Authors:** Conor John McCabe, Uma K. Aryal, Theresa Casey, Jacquelyn Boerman

**Affiliations:** 1 Department of Animal Sciences, Purdue University, West Lafayette, IN 47907, USA; cjmccabe@ucdavis.edu (C.J.M.); jboerma@purdue.edu (J.B.); 2 Purdue Proteomics Facility, Bindley Bioscience Center, Purdue University, West Lafayette, IN 47907, USA; uaryal@purdue.edu; 3 Department of Comparative Pathobiology, Purdue University, West Lafayette, IN 47907, USA

**Keywords:** transition cow, oxidative stress, mitochondrial oxidation

## Abstract

Muscle tissue serves as a key nutrient reservoir that dairy cows utilize to meet energy and amino acid requirements for fetal growth and milk production. Circadian clocks act as homeostatic regulators so that organisms can anticipate regular environmental changes. The objective of this study was to use liquid chromatography tandem mass spectrometry (LC-MS/MS) to determine how chronic circadian disruption in late gestation affected the muscle tissue proteome. At five weeks before expected calving (BEC), multiparous Holstein cows were assigned to either a control (CON, *n* = 8) or a 6 h forward phase shift (PS, *n* = 8) of the light–dark cycle every 3 days. At calving, all animals were exposed to CON light–dark cycles. Muscle biopsies were collected from *longissimus dorsi* muscles at 21 days BEC and at 21 days postpartum (PP). At *p* < 0.1, 116 and 121 proteins were differentially abundant between PS and CON at 21 days BEC and 21 days PP, respectively. These proteins regulate beta oxidation and glycolysis. Between pregnancy and lactation, 134 and 145 proteins were differentially abundant in CON and PS cows, respectively (*p* < 0.1). At both timepoints, PS cows exhibited an oxidative stress signature. Thus, dairy cattle management strategies that minimize circadian disruptions may ensure optimal health and production performance.

## 1. Introduction

Late gestation and early lactation are metabolically demanding physiological states for dairy cattle. The nutrient requirements for early lactation are greater than that for late gestation, because cows require three times more energy to support milk production than the growth of the fetus [1]. Since cows cannot meet these energetic and nutrient demands by increased voluntary feed intake alone, coordinated changes in metabolism occur at the systemic and tissue level to meet these increased requirements [2]. Among the metabolic adaptations is an increase in catabolic processes that results in mobilization of tissue stores to supply amino acids (AA) and lipids as substrates to fuel the growth and development of the fetus and milk synthesis in the mammary glands.

Coordinated changes in metabolism are mediated by the periparturient changes in metabolic and reproductive hormones [1]. One of the metabolic adaptations is reduced insulin sensitivity, which results in decreased tissue deposition and increased fat and protein mobilization. This allows for fatty acid and AA substrates to be transported through the blood to support fetal growth and milk synthesis [3]. Approximately 20% of cows’ protein reserves and 30–35% of their fat reserves are mobilized from late gestation through the first five weeks in lactation [4,5]. Proteomic analysis of periparturient cattle skeletal muscle biopsies using two-dimensional MALDI-TOF analysis has identified 43 differentially abundant proteins between late gestation and early lactation [6]. At the onset of lactation, the muscle proteome is characterized by an increase in the abundance of proteins that promote tissue reserve mobilization such as proteins that regulate fat breakdown, glycolysis, and ATP production. Furthermore, there is a reduced abundance of proteins related to glycogenesis and TCA cycle, thereby, indicating the promotion of tissue reserve mobilization and reduction in tissue storage to meet the early lactation nutrient requirements of the dairy cow.

Although hormones play a key role in regulating the metabolic changes in periparturient cows, several studies have supported a potential role for the circadian timing system (CTS) in mediating adaptions to these physiological states. Metabolic and endocrine systems are integrated and reciprocally regulated by molecular clocks that make up the CTS. The CTS functions to coordinate physiological processes across the body and synchronize behavior and physiology with the external environment. The CTS enables organisms to maintain homeostasis across the 24 h day through generation of circadian rhythms of physiology and behavior in anticipation of regularly occurring events, such as the light–dark cycle. The CTS is organized hierarchically with the master clock located in the suprachiasmatic nuclei (SCN) of the hypothalamus [7]. The SCN receive and integrate internal and environmental temporal cues and, in turn, send out nervous and endocrine signals to peripheral clocks that are distributed in every cell of the body [8]. Tissue clocks generate circadian rhythms of gene expression related to organ function. Alterations in the timing of environmental events can disrupt circadian clocks. Chronic disruption of circadian clocks affects metabolism and is associated with the development of insulin resistance and a prediabetic state in multiple species, including dairy cows [9,10,11].

We hypothesized that circadian clocks play a role in mediating metabolic adaptations that occur in the periparturient period, and thus speculate that CTS disruption in late gestation through exposure of cattle to chronic light–dark phase shifts would affect the periparturient metabolic adaptations in muscle tissue. The objective of this study was to use liquid chromatography tandem mass spectrometry (LC-MS/MS) to determine how chronic circadian disruptions in late gestation affected the muscle tissue proteome. Furthermore, we investigated whether there was a carry-over effect from prepartum circadian system disruption on muscle proteome at three weeks post-parturition.

## 2. Materials and Methods

### 2.1. Animal Management and Experimental Design

**Figure 1:**
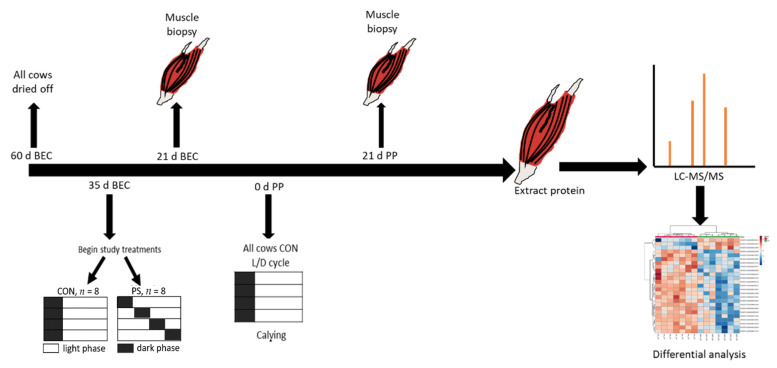
Graphical schematic of the experimental design. Dairy cattle were dried off (milking was ceased) at 60 days before expected calving (BEC). At 35 days BEC cows were moved into a tie-stall barn and exposed to a control (CON, *n* = 8) or forward 6 h phase shift (PS, *n* = 8) of the light–dark cycle every three days. At calving, all animals were exposed to the CON light–dark cycles through 60 days postpartum (PP). *Longissimus dorsi* (LD) muscle was biopsied at 21 days BEC and 21 days PP. Proteins were extracted from LD biopsy samples and analyzed via liquid chromatography tandem mass spectrometry (LC-MS/MS). Multivariate and univariate statistical tests were used to identify proteins differentially abundant between treatments and timepoints.

The experiment was performed in the tie-stall barn at the Purdue University Animal Science Research and Education Center (ASREC) for dairy. All procedures described were approved by IACUC protocol #1701001523 prior to beginning any experiments. Sixteen multiparous Holstein cows were blocked to two treatments, either control (CON) or phase shift (PS) by lactation number (2.88 ± 0.64 vs. 2.88 ± 0.64) previous 305-day lactation yield (12,087 ± 2486 vs. 12,467 ± 2407 kg), and metabolic disease during the previous lactation (ketosis, displaced abomasum, mastitis, or milk fever). Cows were enrolled to treatment at 35 days BEC to either a control (CON, *n =* 8), light–dark cycle of 16 h of light and 8 h of dark, or a 6 h phase shift (PS, *n =* 8) at the start of the light–dark phase cycle every three days. At the time of calving, all cows received the CON light–dark cycle. Cows were fed a standard late gestation diet during the period prior to calving and a lactating diet post-calving to meet nutrient requirements (NRC, 2001). The prefresh diet consisted predominately of corn silage, straw, and additional by-products, whereas the lactating diet consisted of alfalfa hay, corn silage, and alfalfa haylage. The feed was delivered to all cows for an expected 10% refusals at ~1600 h. Once animals calved, they were milked twice each day at 0500 h and 1600 h. Cows exposed to the PS treatment produced less milk per day as compared with the CON, through 60-DIM. Details of the barn, separation of treatments for light exposures and shifting phases, and study outcomes have been described in extensive detail in our previous publication [11]. A graphical schematic of the experimental design is shown in Figure 1.

At 21 days BEC and 21 days PP, *longissimus dorsi* biopsies were performed at approximately 0700 h. The *longissimus dorsi* muscle was chosen, as changes in its depth and composition in relation to nutritional manipulations and physiological states of animal have been well characterized [12,13,14]. Biopsies taken at 21 days BEC were collected between the 9th and 12th intercostal spaces on the left side of the animal. The subsequent biopsy at 21 days PP was performed one intercostal space in the cranial direction. On the day of the biopsy, the animal was restrained within a livestock handling chute (Tarter Farm & Ranch Equipment, Dunnville, KY, USA). Cattle were sedated with xylazine hydrochloride (35–45 µg/kg of body weight, Rompun, Bayer HealthCare LLC, Animal Health Division, Shawnee, Kansas). Ampicillin was also administered at a dose of 10 mg/kg approximately 10 min prior to biopsy. The procedure site was shaved and cleaned with a 7.5% betadine scrub (Vet One, Boise, ID, USA) and 70% isopropyl alcohol (Vet One, Boise, ID, USA) within 10 min of local anesthetic injection. Five mL of lidocaine (Vet One, Boise, ID, USA) was administered at the injection site two minutes prior to incision. A one-inch horizontal incision was made through the skin with a scalpel blade (Vet One, Boise, ID, USA) to cut through the skin and fascia to expose muscle. The muscle biopsy was performed with a Bergstrom needle (AgnTho’s, Lidingö, Sweden) using a circular motion to capture a representative sample. Biopsy cuts were performed at six unique points around the incision site to excise a core of ~1.0 g of representative muscle tissue. Then, the muscle tissue was removed from the needle into a small amount of sterile phosphate buffered saline (PBS) in a cell culture grade Petri dish before it was split into two aliquots and placed into cryovials, and flash frozen in liquid nitrogen. The samples remained frozen at −80 °C until protein isolation.

### 2.2. Sample Preparation for Proteomic Analysis

Protein sample preparation and shotgun liquid chromatography tandem mass spectrometry (LC-MS/MS) analysis was performed at Purdue University’s Proteomics Core. To start, frozen tissues were thawed and homogenized in 100 mM ammonium bicarbonate buffer at 6500 rpm for 90 s in a bead beater (Bertin Technologies SAS, Paris, France). Then, the homogenate was transferred to a new tube, and protein concentration was calculated using a bicinchoninic assay (BCA, Thermo Fisher Scientific, Waltham, MA, USA). Fifty µg of protein was precipitated overnight, using 1:4 volume of cold (−20 °C) acetone. After overnight precipitation, the protein was pelleted by centrifugation at 13,500 rpm for 15 min at 4 °C. Subsequently, protein pellets were dried and dissolved in 8 M urea containing 10 mM dithiothreitol (DTT) and incubated at 37 °C for 1 h for disulfide bond reduction. Next, cysteines were alkylated by incubating the sample with an alkylating reagent mix consisting of 97.5% acetonitrile, 0.5% triethylphosphine, and 2% of iodoethanol for one h in the dark at room temperature. The samples were dried in a vacuum centrifuge. A trypsin/LysC combination (Promega, Madison, WI, USA) was used for sample digestion. The manufactured trypsin/LysC vial was dissolved in 400 µL of 50 mM ammonium bicarbonate, and 80 µL of the enzyme was added to each sample. Digestion was performed at high pressure using a Barocycler (50 °C, 60 cycles, 20,000 psi, Pressure Biosciences, Easton, MA, USA). Digested peptides were desalted using MicroSpin columns (C18 silica, The Nest Group Inc., Ipswich, MA, USA). Purified peptides were heated in a vacuum centrifuge and stored at −80 °C until analysis. Purified peptides were resuspended in 3% acetonitrile/0.1% formic acid to a final concentration of 1 µg/µL and 1 µL was used for LC-MS/MS analysis.

### 2.3. LC-MS/MS Analysis for Peptide Sequencing

Peptides were analyzed in an Dionex UltiMate 3000 RSLC nano System (Thermo Fisher Scientific, Odense, Denmark) coupled on-line to an Orbitrap Fusion Lumos Mass Spectrometer (Thermo Fisher Scientific, Waltham, MA, USA), as described previously [15]. Two solvents were made for analysis with solvent A, which constituted 0.1% formic acid in water and solvent B made of 80% acetonitrile, 19.99% water, and 0.1% formic acid. Briefly, reverse phase peptide separation was accomplished using a trap column (300 μm ID × 5 mm) packed with 5 μm 100 Å PepMap C18 medium coupled to a 50 cm long × 75 µm inner diameter analytical column packed with 2 µm 100 Å PepMap C18 silica (Thermo Fisher Scientific). The column temperature was maintained at 50 °C. A sample was loaded for 5 min to the trap column and eluted from the analytical column at a flow rate of 200 nL/min using a 160 min LC gradient. Analysis was carried out in the following order: a linear gradient from 2 to 27% of solvent B for 110 min, 27–40% of B for the next 15 min, 40–100% of B for the next 10 min, at which point the gradient was held at 100% of B for 10 min, before reverting back to 2% of B, and held at 2% of B for 10 min for column equilibration. The column was further washed and equilibrated by using three 30 min LC gradient periods before injecting the next sample. All data were acquired in the Orbitrap mass analyzer using an HCD fragmentation scheme. For MS scans, the scan range was from 350 to 1600 m/z at a resolution of 120,000. The automatic gain control (AGC) target was set at 4 × 105, maximum injection time 50 ms, dynamic exclusion 30 s, and intensity threshold 5.0 × 104. MS data were acquired in data dependent mode with cycle time of 5 s/scan. MS/MS data were collected at a resolution of 15,000.

### 2.4. Data Analysis

LC-MS/MS data were mapped to peptides using MaxQuant software (version 1.6.3.3) against Bos Taurus sequence database downloaded from the UniProt (www.uniprot.org; v. 2020_05, accessed on 20 May 2020). For each search, the following parameters were used: precursor mass tolerance of 10 ppm, enzyme specificity of trypsin/Lys-C enzyme allowing up to 2 missed cleavages, oxidation of methionine (M) as a variable modification, and iodoethanol (C) as a fixed modification. Data were filtered to retain only proteins with LFQ > 0 and MS/MS (spectral counts) ≥ 2 for additional analysis. One animal from the PS treatment and one animal from the CON group had their postpartum sample removed prior to analysis due to poor sample quality. Raw LC-MS/MS data were made publicly available by depositing in the MassIVE (https://massive.ucsd.edu, accessed on 18 May 2021) database and are available through the following accession number: MSV000087432.

The label-free quantification (LFQ) of the protein was used for analysis of differential abundance between the treatments and timepoints. To be retained for analysis, each protein had to be detected in at least three animals in each treatment or timepoint of interest. Protein UniProt IDs were converted into Ensembl IDs using the NIH Database for Annotation, Visualization, and Integrated Discovery (DAVID) v6.8 (https://david.ncifcrf.gov/tools.jsp; accessed on 16 June 2020). All LFQ measurements were log_2_ transformed to create a normal distribution of protein abundance. Student t-tests were performed to compare treatments within timepoints at 21 days BEC and 21 days PP, and physiological states within CON and PS treatments using Metaboanalyst (https://www.metaboanalyst.ca; accessed on 9 June 2020). A difference between treatment and timepoint was considered to be differently abundant using the nominal *p*-value of <0.10. DAVID, PANTHER (http://www.pantherdb.org/, accessed on 16 June 2020), Qiagen Ingenuity Pathway Analysis (https://digitalinsights.qiagen.com/products-overview/discovery-insights-portfolio/analysis-and-visualization/qiagen-ipa/; accessed on 29 June 2021), GeneCards (https://www.genecards.org/, accessed on 16 June 2020), and UniProt (https://www.uniprot.org/, accessed on 20 May 2020) databases were used for functional annotation analysis of differentially abundant proteins between timepoints and treatments.

## 3. Results

### 3.1. Number of Proteins Differentially Abundant

Analysis of the relationship of log base 2 of protein LFQ intensity among samples demonstrated strong correlation coefficients among samples from cows within the same treatment and timepoints (r ≥ 0.94, Figure S1). The retained samples (*n* = 8 prepartum CON, *n* = 8 prepartum PS, *n* = 7 postpartum CON, and *n* = 7 postpartum PS) and LC-MS/MS data acquisition were suitable for downstream analysis and showed strong similarities among animals between treatments and timepoints.

**Figure 2:**
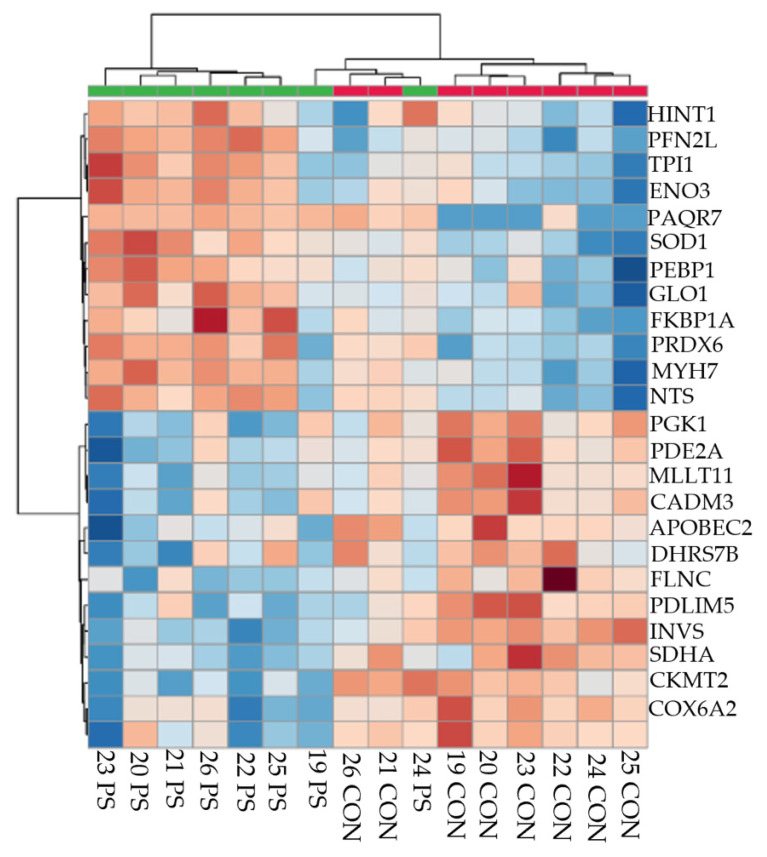
Heatmap and dendrogram of hierarchical cluster analysis showing top 25 differentially abundant *longissimus dorsi* proteins between control (CON, red, *n* = 8) and 6 h forward phase shift (PS, *n* = 8) of the light–dark cycle cattle.

Searching against the bovine UniProt database returned 888 proteins (IDs) that mapped to the LC-MS/MS spectra across the 30 samples (Table S1). A protein was retained for downstream analysis within a treatment-timepoint when at least three animals from a treatment or timepoint expressed that protein (Supplementary Table S2A–D). Hierarchical and principal component analysis of these sets of proteins indicated that physiological state (3 weeks BEC versus 3 weeks PP) and treatment (CON versus PS) affected protein expression profiles (Figure 2). The analysis of the number of differentially abundant proteins among comparisons by *p*-value, found that relaxing the stringency of the nominal *p*-value from 0.01 to 0.1 increased the number of proteins to consider for functional annotation analysis from as few as 3 to as many as 126 (Table 1). Thus, proteins passing the *p*-value filter of <0.1 were used for downstream analysis.

### 3.2. Treatment Comparisons

**Figure 3:**
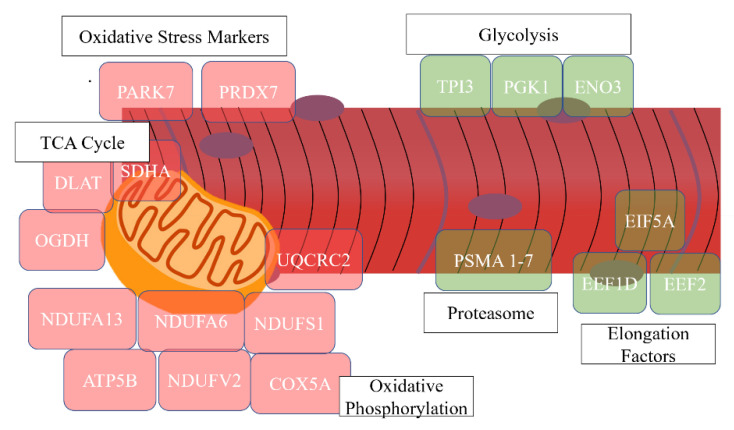
Graphical representation of differentially abundant *longissimus dorsi* proteins between control (CON, *n* = 8) and phase shifted (PS, *n* = 8) cattle at 21 days before expected calving (BEC). Proteins are grouped based on the functional groups in muscle tissue. Proteins highlighted in red are of greater abundance in PS as compared with the CON cattle, whereas proteins highlighted in green are of lesser abundance in PS versus CON cattle.

At 21 days BEC, 74 (15.7%) of the 470 proteins were differentially abundant between PS and CON cows (Table 2). In PS relative to CON cows, there was a lower abundance of proteins related to translation activity (10%), proteasome (10%), and glycolysis (7%) (Table 2 and Figure 3). Translation proteins lower in abundance included the elongation factors of EIF5A, EEF1D, EEF2, and EEF1A2. In the proteasome category, PS cows had a lower abundance of four of the seven subunits of the proteasome (PSMA1–3 and PSMA 7). The glycolytic enzymes lower in PS than in CON cows were triosephosphate 1 (TPI1), phosphoglycerate kinase 1 (PGK1), and enolase 3 (ENO3). The PS cows had a greater abundance of proteins at three weeks BEC than CON that enriched categories related to oxidative phosphorylation (19%), actin binding (12%), and the tricarboxylic acid (TCA) cycle (4%). These proteins included several subunits of the NADH: ubiquinone oxidoreductase core subunits (NDUFS1, NDUFA6, NDUFA13, NDUFV2, and NDUFA9) that make up complex I of the electron transport chain. In addition, more abundant in the *longissimus dorsi* muscle of PS cows at three weeks BEC were succinate dehydrogenase (SDHA), oxoglutarate dehydrogenase (OGDH), and dihydrolipoamide S-acetyltransferase (DLAT), which all play a role in ATP generation in the TCA cycle. Additionally, among the proteins more abundant in muscle of PS cattle at this timepoint were two proteins that mediate response to oxidative stress, i.e., Parkinsonism associated deglycase 7 (PARK7) and peroxiredoxin-6 (PRDX6).

Similarly at 21 days PP, proteins more abundant in PS cows had enriched pathways related to oxidative phosphorylation (10%), the TCA cycle (10%), and fatty acid beta oxidation (4%). Proteins in these categories were related to ATP synthesis (ATP5B, ATP5J2, ATP5H, ATP5I, ATP5J, and ATP5F1), the electron transport chain (NDUFA9, NDUFS1, and NDUFS3), and redox reactions with several dehydrogenases involved in the conversation of substrates to ATP (GAPDH, LDHA, LDHB, PDHB, and MDH1). There was also an increased abundance of proteins related to the heat shock proteins (HSPA8, HSPA9, and HSPA1A) in the PS as compared with the CON cows at 21 days PP.

### 3.3. Physiological State Comparisons

**Figure 4:**
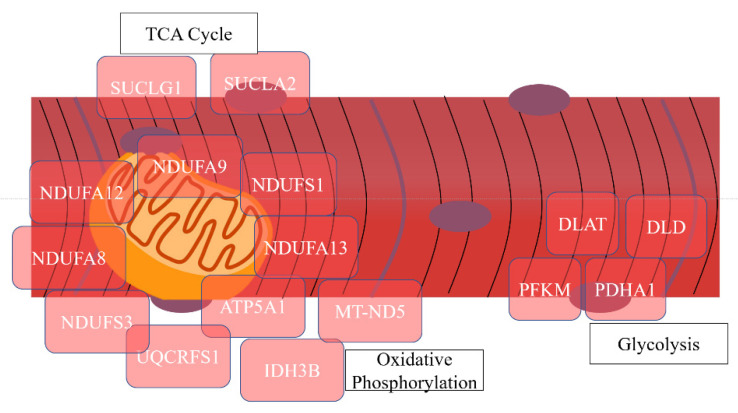
Graphical representation of differentially abundant *longissimus dorsi* proteins between 21 days before expected calving (BEC, *n* = 8) and 21 days postpartum (PP, *n* = 8) timepoints in Control (CON) cattle. Proteins are grouped based on functional groups in muscle tissue. Proteins highlighted in red are of greater abundance at 21 days PP than at 21 days BEC.

In CON cows during the physiological adaptation from 21 days BEC to 21 days PP, there was a decrease in the abundance of nucleosome core proteins that are involved in histone modification (H2AFV, HIST2H2BE, and H3F3A). Whereas proteins related to oxidative phosphorylation (25%), the TCA cycle (9%), and ATP binding (14%) increased from 3 weeks BEC to 21 days PP (Figure 4). Proteins that enriched oxidative phosphorylation included NDUFA12, NDUFA9, NDUFS1, NDUFA7, and NDUFA5, and proteins that enriched the TCA cycle included SUCLG1, CS, IDH3B, and SUCLA2, among other enzymes involved in energy metabolism.

In PS cows, there were 19 proteins that decreased in abundance in muscle from late gestation to three weeks PP. The functional annotation cluster analysis with DAVID did not return any categories reflective of protein function. Proteins that decreased were histone H3 and H1 cluster proteins, as well as ubiquitin B. Proteins that increased in abundance between 3 weeks prepartum and 3 weeks postpartum enriched the TCA cycle (7%), oxidative phosphorylation (17%), and ATP synthesis (3%). These proteins included four subunits of ATP synthase (ATP5H, ATP5I, ATP5J, and ATP5F1) and subunits of succinate dehydrogenase (SUCLA2 and SUCLG1). There was also an increase in abundance of proteins that regulate fatty acid degradation (5%) and glycolysis and gluconeogenesis (13%). Genes of interest in these categories included ALDH2, ALDOA, and TPI1, which are involved in the breakdown of glucose and gluconeogenic substrates.

## 4. Discussion

Two weeks of exposure to chronic light–dark phase shifts significantly impacted the muscle proteome. Profiles of PS cows were indicative of greater mitochondrial oxidation at both timepoints measured. Differences in muscle proteomes of cows in early lactation versus late gestation were also characterized by an increase in abundance of proteins that regulate mitochondrial oxidative phosphorylation, and the breakdown of fatty acid and glucose substrates. The transition from late gestation to early lactation is marked by an almost two-fold increase in AA requirements along with a three-fold increase in energy demands [16]. Dairy cattle mobilize muscle tissue around the time of calving through to at least 5 weeks postpartum due to insufficient dry matter intake and heightened AA demands of milk production [17]. In response to the increased energy demands of lactation, mitochondria metabolic output in muscle is increased [18]. The increase in abundance of proteins that regulate glycolysis indicate that muscle plays a role in supplying glucose to the mammary glands to support their increased nutrient requirements during the early lactation period [19]. Thus, changes in muscle proteome signatures from late gestation to early lactation reflect adaptations in muscle that occur so that cows can meet the increased energetic and substrate demands of lactation. These metabolic adaptations are regulated by a decrease in peripheral tissue insulin sensitivity [20]. Our previous study found that exposure to three weeks of chronic light–dark phase shifts (35–14 days BEC) decreased insulin sensitivity of late gestation cows [11]. Despite cessation of exposure to phase-shifting light–dark cycle around the time of parturition, a greater degree of insulin resistance in PS versus CON cows was carried over into the postpartum. Thus, the greater abundance of proteins that regulate mitochondrial oxidative phosphorylation, and the breakdown of fatty acid and glucose substrates in PS cows likely reflect the decrease in insulin sensitivity in these cows.

At 21 days BEC and 21 days PP, muscle proteome of PS cows demonstrated signatures that indicated greater levels of energy generation as compared with their CON counterparts with an increased abundance of proteins associated with oxidative phosphorylation and the TCA cycle. Higher levels of cellular oxidative phosphorylation are balanced by increased expression of antioxidants to prevent oxidative stress, which can negatively impact mitochondrial function [21]. The transition from gestation to lactation is the greatest period of metabolic stress in the lactation cycle [22]. While this period is characterized by increased oxidative stress, a link has also been established between circadian disruptions and oxidative stress in other species [23]. In addition to the proteins represented as increased in abundance in PS cows at 21 days BEC in Table 2, PARK7 and PRDX6 were both elevated. These proteins function as antioxidants that prevent buildup of oxidative species [24]. PS cows also exhibited an increased abundance of HSP70 at 21 days PP. Heat shock proteins function as chaperones that stabilize new proteins and refold proteins damaged by cellular stress. Heat shock protein abundance during cellular stress response enables the maintenance of cell function. Heat shock proteins are found to be elevated in heat-stressed dairy cows [25], and thus higher levels of heat shock proteins and antioxidant proteins indicate that PS cows were under greater levels of metabolic stress due to circadian disruption. Cattle that experience greater oxidative stress exhibit reduced milk production in early lactation [26]. In our study PS cows produced significantly less milk than CON cows through 60 days post parturition [11]. Therefore, oxidative stress at the level of the muscle tissue may have impacted the cows’ abilities to coordinate the metabolic changes necessary for early lactation milk production.

Previous studies have shown that cows with greater oxidative stress have a greater abundance of proteins involved in the ubiquitin proteasome pathway [27]. This pathway is responsible for removing toxins and recycling wastes from cells and tissues. The ubiquitin proteasome pathway regulates and is reciprocally regulated by the circadian timing system [28]. The ubiquitin proteasomal pathway is a key regulator of the degradation of the core clock components PER and CRY, which in turn relieves their inhibition of BMAL1:CLOCK activity [29]. Transgenic mice with homozygous knockout of *Bmal1* exhibit signs of premature aging related to the buildup of free radicals in cells [30]. Progressive aging is marked by the buildup of damaged proteins that result partially from oxidative damage. Thus, the proteome signature of PS-treated cattle may reflect the effects of circadian clock disruption [28].

In the same vein, PS cows also had a decreased abundance of eukaryotic elongation factor proteins during the prepartum period. Studies of fungi have linked 24 h periodicity of mRNA translation to circadian clock regulation of EEF-2 [31], with many of the regulatory mechanisms conserved across diverse species [32]. More than 2300 genes have been found to exhibit circadian rhythms of gene expression in muscle tissue, and function in the regulation of transcription, myogenesis, and metabolism [9]. Skeletal muscle circadian rhythms are entrained to light input through signal from the master clock in the SCN as well as temporal information regarding time of feeding and activity. Circadian rhythm disruption is related to muscle fiber-type shifts, altered sarcomeric structure, reduced mitochondrial respiration, and impaired muscle function.

To maximize milk production, the health and welfare of cattle must be considered in management practices of dairy systems [33]. Chronic shifting of light–dark cycles is not an environment that dairy cattle will likely ever be exposed to in their lifetime. However, the chronic jet-lag paradigm used enables the probing of the fundamental biology of the cow, and the data analysis of the effects of exposure provides insight into the role of clocks in regulation of physiology during late gestation. Light, stress, exercise, and nutrition affect dairy cow welfare and are inputs to the circadian timing system [34]. Management systems that disrupt 24 h cycles of these inputs can potentially disrupt circadian clocks. As a component of the homeostatic system, chronic disruption of clocks appears to negatively impact metabolic balance at the systemic and tissue level. As mentioned here, protein signatures of PS-treated cattle indicated greater metabolic-oxidative stress in skeletal muscle in response to environmental disruptors. Therefore, dairy farmers should look to reduce disruptors to the circadian rhythm to minimize the impacts on milk production and muscle tissue health as cows transition from late gestation to early lactation. This transition to lactation has much greater nutrient requirements than the gestational period and cows must be fed and managed accordingly to meet these nutrient needs.

## 5. Conclusions

Dairy cows exposed to continuous light–dark phase shifts during the late gestational period had muscle proteome signatures indicative of increased oxidative phosphorylation, fatty acid breakdown, and oxidative stress. Despite the cessation of phase-shift treatment at calving, this effect was carried over to 21 days postpartum. As cows increased in energetic demands from late gestation to early lactation, they experienced an increased abundance of proteins related to oxidative output and gluconeogenesis. Understanding muscle proteomic signatures around the calving period in relation to outside stressors can identify intervention opportunities to overcome the observed negative milk production and metabolic and tissue health impacts associated with management strategies that may disrupt the circadian timing system.

## Figures and Tables

**Table 1:** Number of proteins differentially abundant between control (CON, *n* = 8) and phase shifted (PS, *n* = 8) treatment cattle and number of differentially abundant proteins between timepoints of 3 weeks before expected calving (BEC, *n* = 16) and 3 weeks postpartum (PP, *n* = 14). Differences are represented at the α = 0.01, 0.05, and 0.10 levels.

Proteins Differentially Abundant between CON and PS Cows at the Prepartum and Postpartum Timepoints							
	Proteins per Comparison	Lower Abundance in PS			Greater Abundance in PS		
***p*** **-Value Cutoff**	**-**	***p*** **< 0.01**	***p*** **< 0.05**	***p*** **< 0.10**	***p*** **< 0.01**	***p*** **< 0.05**	***p*** **< 0.10**
3 weeks BEC	470	13	29	42	13	52	74
3 weeks PP	312	3	3	8	22	72	113
**Proteins Differentially Abundant between Prepartum and Postpartum Timepoints within Treatments**							
	**Proteins per Comparison**	**Lower Abundance in PP**			**Greater Abundance in PP**		
***p*** **-Value Cutoff**	**-**	***p*** **< 0.01**	***p*** **< 0.05**	***p*** **< 0.10**	***p*** **< 0.01**	***p*** **< 0.05**	***p*** **< 0.10**
CON treatment	462	10	26	42	25	64	92
PS treatment	315	6	12	19	50	103	126

**Table 2:** Functional annotation clusters resulting from DAVID analysis significantly enriched with proteins differentially abundant between control (CON, *n* = 8) and phase shifted (PS, *n* = 8) cattle at the physiological timepoints of 3 weeks before expected calving (BEC) and 3 weeks postpartum (PP), *n* = 7/treatment. Enriched clusters of proteins differentially abundant between the BEC and PP physiological states within CON and PS treatments.

Lower Abundance in PS versus CON at 3 weeks BEC				
Category	Number	Percent	*p*-Value	Proteins
Proteasome	4	10.26	1.45 × 10^−4^	PSMA1, PSMA2, PSMA3, PSMA7
Translation elongation factor activity	4	10.26	1.51× 10^−5^	EIF5A, EEF1D, EEF2, EEF1A2
Glycolysis	3	7.69	0.0013	TPI1, PGK1, ENO3
**Greater Abundance in PS versus CON at 3 weeks BEC**				
**Category**	**Number**	**Percent**	***p*-Value**	**Proteins**
Oxidative phosphorylation	14	20.00	1.97 × 10^−13^	ATP5B, NDUFV2, NDUFA9, SDHA, NDUFS1, UQCRFS1, UQCRC2, NDUFA13, NDUFA6, COX5A, ATP5O, COX5B, COX6A2, CYC1
Skeletal muscle contraction	5	7.14	8.92 × 10^−7^	TNNT1, TNNI1, MYH7, TNNC1, TNNI2
Actin-binding	9	12.86	4.21 × 10^−8^	MYH4, TPM2, ACTN4, MYH7, ACTN1, MYH2, TPM3, FLNC, ACTN2
Tricarboxylic acid cycle	3	4.29	0.0062	SDHA, OGDH, DLAT
**Lower Abundance in PS versus CON at 3 weeks PP**				
**Category**	**Number**	**Percent**	***p*-Value**	**Proteins**
None				
**Greater abundance in PS at 3 weeks PP**				
**Category**	**Number**	**Percent**	***p*-Value**	**Proteins**
Tricarboxylic acid cycle	11	10.68	4.97 × 10^−16^	MDH1, SUCLG1, CS, PDHB, ACO2, SDHB, SDHA, FH, OGDH, DLST, PDHA1
Glycolysis/gluconeogenesis	13	12.62	4.71 × 10^−13^	PGAM2, GAPDH, LDHA, PDHB, ALDH2, GPI, PFKM, TPI1, PGK1, PKM, LDHB, FBP2, PDHA1
Oxidative phosphorylation	11	10.68	8.00 × 10^−7^	NDUFS3, ATP5B, ATP5J2, ATP5H, NDUFA9, NDUFS1, SDHB, SDHA, ATP5I, ATP5J, ATP5F1
Fatty acid beta-oxidation	4	3.88	0.0012	ACAA2, ACAT1, HADHB, HADHA
Nucleotide phosphate-binding region:NAD	7	6.80	1.83 × 10^−5^	MDH1, GAPDH, LDHA, NNT, ALDH2, LDHB, ALDH1A1
Electron transport	6	5.83	1.44 × 10^−4^	NDUFS3, NDUFA9, ETFA, NDUFS1, SDHB, SDHA
Heat shock protein 70	3	2.91	0.0030	HSPA8, HSPA9, HSPA1A
**Proteins that Decreased in Abundance between 3 weeks BEC and 3 weeks PP in CON Cows**				
**Category**	**Number**	**Percent**	***p*-Value**	**Proteins**
Nucleosome core	3	8.11	0.015	H2AFV, HIST2H2BE, H3F3A
**Proteins that Increased in Abundance between 3 weeks BEC and 3 weeks PP in CON Cows**				
**Category**	**Number**	**Percent**	***p*-Value**	**Proteins**
Oxidative phosphorylation	23	26.14	5.76 × 10^−22^	NDUFA12, NDUFA9, NDUFS1, NDUFA7, NDUFV1, UQCRFS1, ATP5F1, NDUFA8, NDUFB10, ND5, NDUFA5, UQCRC2, NDUFS3, NDUFA13, NDUFA6, COX4I1, COX5A, ATP5O, ATP5A1, COX5B, COX6A2, CYC1
NAD	11	12.50	3.58 × 10^−10^	NDUFS3, NNT, IDH3B, VDAC2, NDUFS1, NDUFV1, GPD1, IDH3A, DLD, MT-ND5, VDAC1
Mitochondrial respiratory chain complex IV	5	5.68	7.42 × 10^−7^	NDUFA4, COX5A, COX4I1, COX5B, COX6A2
Tricarboxylic acid cycle	8	9.09	7.68 × 10^−12^	SUCLG1, CS, IDH3B, SUCLA2, IDH2, IDH3A, PDHA1, DLAT
Glycolysis/gluconeogenesis	4	4.55	0.020	DLD, PFKM, PDHA1, DLAT
ATP-binding	13	14.77	7.49 × 10^−4^	OBSCN, MYLK2, MYH4, PHKG1, HSPA9, PFKM, AP2A1, COQ8A, CKMT2, SUCLA2, VCP, MYH2, ATP5A1
**Proteins that Decreased in Abundance between 3 weeks BEC and 3 weeks PP in PS Cows**				
**Category**	**Number**	**Percent**	***p*-Value**	**Proteins**
None				
**Proteins that Increased in Abundance between 3 weeks BEC and 3 weeks PP in CON Cows**				
**Category**	**Number**	**Percent**	***p*-Value**	**Proteins**
Glycolysis/gluconeogenesis	16	13.22	1.18 × 10^−15^	PGAM2, GAPDH, LDHA, PDHB, ALDH2, ALDOA, ENO3, GPI, PFKM, PGM1, TPI1, PGK1, PKM, LDHB, FBP2, PDHA1
Tricarboxylic acid cycle	9	7.44	8.65 × 10^−13^	SUCLG1, MDH2, CS, PDHB, ACO2, SUCLA2, SDHB, DLST, PDHA1
Oxidative phosphorylation	21	17.36	5.53 × 10^−16^	NDUFA4, ATP5B, ATP5J2, ATP5H, NDUFA9, NDUFS1, SDHB, NDUFV1, ATP5F1, NDUFA10, NDUFB10, NDUFA5, NDUFS2, NDUFS3, UQCRB, NDUFA6, UQCRC1, COX4I1, COX5A, ATP5I, ATP5J
NAD	14	11.57	1.45 × 10^−16^	HSD17B10, GAPDH, LDHA, NNT, MDH2, VDAC2, DHRS7B, ALDH2, NDUFS1, NDUFV1, NDUFS2, NDUFS3, GPD1, LDHB
Mitochondrial proton-transporting ATP synthase complex	4	3.31	3.18 × 10^−5^	ATP5H, ATP5I, ATP5J, ATP5F1
Fatty acid degradation	7	5.79	1.28 × 10^−5^	HADH, ACAT1, ACADVL, ACSL1, HADHB, HADHA

## Data Availability

Raw LC-MS/MS data were made publicly available by depositing them in the MassIVE (https://massive.ucsd.edu, accessed on 18 May 2021) database and are available through the following accession number: MSV000087432.

## Supplementary Materials

The following are available online at https://www.mdpi.com/article/10.3390/proteomes9030035/s1, Table S1: Raw proteomic label free quantification (LFQ) for all 888 abundant proteins. Data output was log_2_ transformed to create a normal distribution. Table S2A: Label free quantification (LFQ) protein abundance comparison between control (CON; *n* = 8) and phase shifted (PS; *n* = 8) dairy cows at 21 days before expected calving (BEC). Table S2B: Label free quantification (LFQ) protein abundance comparison between control (CON; *n* = 7) and phase shifted (PS; *n* = 7) dairy cows at 21 days postpartum (PP). Table S2C: Label free quantification (LFQ) protein abundance comparison between 21 days before expected calving (BEC; *n* = 8) and 21 days postpartum (PP; *n* = 7) for control (CON) dairy cows. Table S2D: Label free quantification (LFQ) protein abundance comparison between 21 days before expected calving (BEC; *n* = 8) and 21 days postpartum (PP; *n* = 7) for phase shifted (PS) dairy cows. Figure S1: Correlation analysis of label free quantification (LFQ) abundance with log_2_ transformation between samples within the same timepoint (21 days before expected calving (BEC) or 21 days postpartum (PP)) and treatment (Control (CON) or Phase Shift (PS)), R^2^ ≥ 0.94.
