# Supplementary material for: Impact of Exposure to Chronic Light–Dark Phase Shifting Circadian Rhythm Disruption on Muscle Proteome in Periparturient Dairy Cows
Source: Proteomes. 2021 Jul 29;9(3):35. doi: 10.3390/proteomes9030035 (PMC8396217; doi:10.3390/proteomes9030035)
Supplement: Supplementary file 1 [file proteomes-09-00035-s001.zip › Supp Fig 1.pdf]

Supplementary Figure S1A.

	19PS_PP	21PS_PP	22PS_PP	23PS_PP	24PS_PP	25PS_PP	26PS_PP
19PS_PP	1	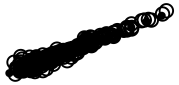	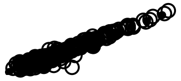	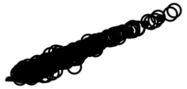	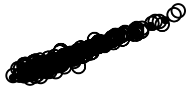	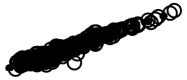	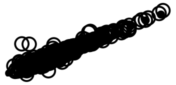
21PS_PP	0.97	1.00	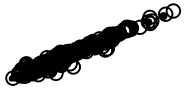	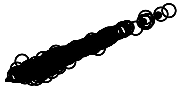	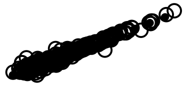	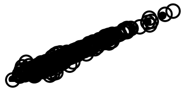	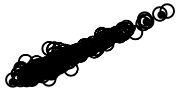
22PS_PP	0.97	0.98	1.00	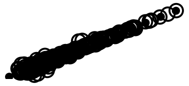	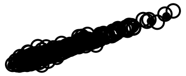	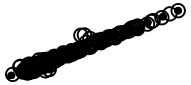	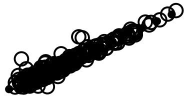
23PS_PP	0.98	0.98	0.99	1.00	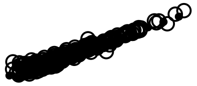	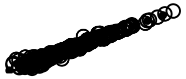	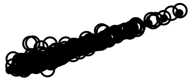
24PS_PP	0.97	0.97	0.97	0.97	1.00	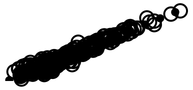	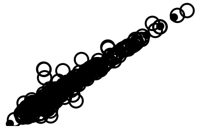
25PS_PP	0.97	0.97	0.99	0.98	0.97	1.00	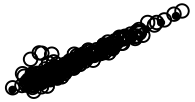
26PS_PP	0.96	0.96	0.96	0.96	0.97	0.96	1.00

Supplementary Figure S1B.

	19c_PP	*21c_PP*	*22c_PP*	*23c_PP*	*24c_PP*	*25c_PP*	*26c_PP*
19c_PP	1.00	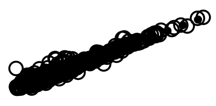	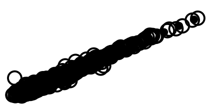	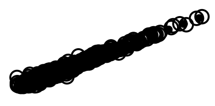	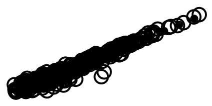	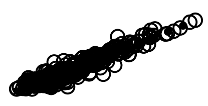	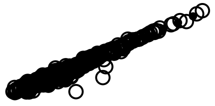
21c_PP	0.97	1.00	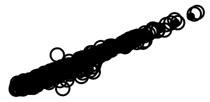	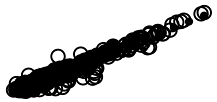	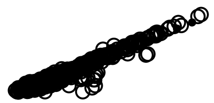	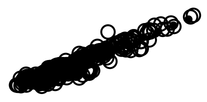	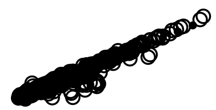
22c_PP	0.99	0.98	1.00	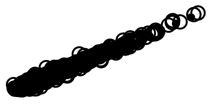	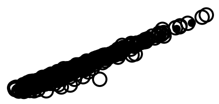	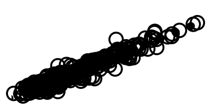	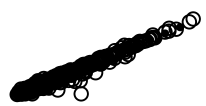
23c_PP	0.99	0.97	0.99	1.00	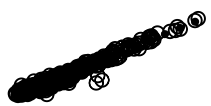	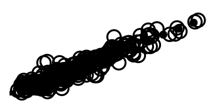	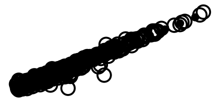
24c_PP	0.97	0.96	0.98	0.98	1.00	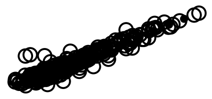	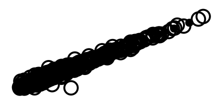
25c_PP	0.95	0.94	0.96	0.94	0.96	1.00	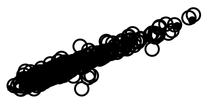
26c_PP	0.98	0.96	0.98	0.98	0.98	0.95	1.00

Supplementary Figure S1C.

	19ps_BEC	20ps_BEC	21ps_BEC	22ps_BEC	23ps_BEC	24ps_BEC	25ps_BEC	26ps_BEC
19ps_BEC	1.00	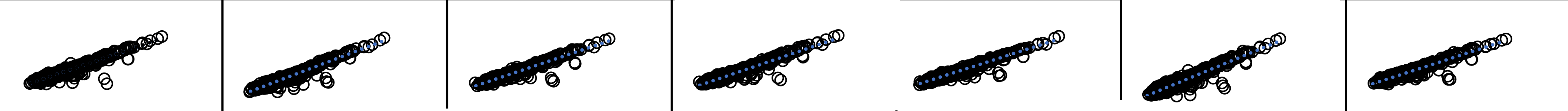						
20ps_BEC	0.95	1.00	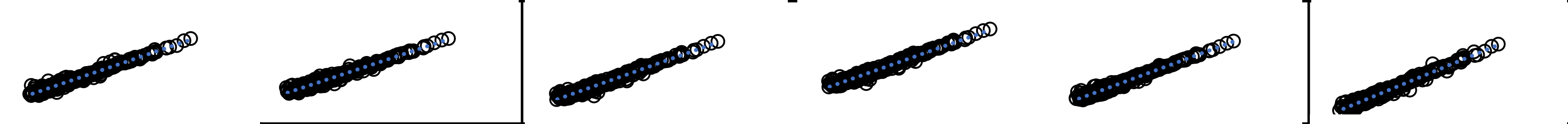					
21ps_BEC	0.96	0.99	1.00	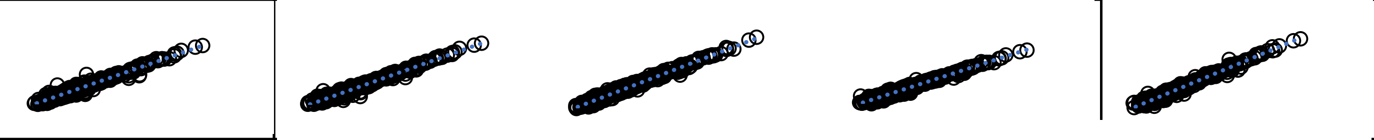				
22ps_BEC	0.96	0.99	0.98	1.00	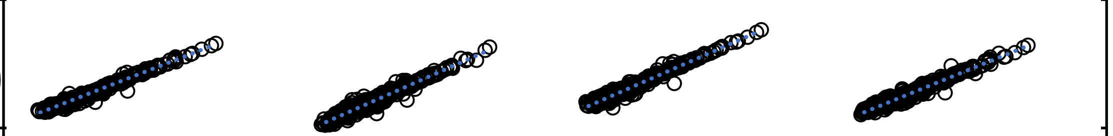			
23ps_BEC	0.95	0.99	0.99	0.99	1.00	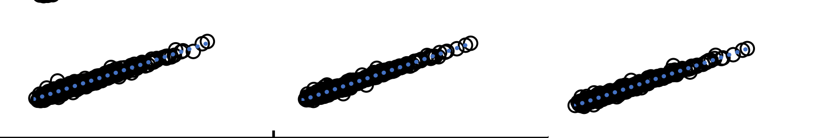		
24ps_BEC	0.96	0.99	0.99	0.98	0.98	1.00	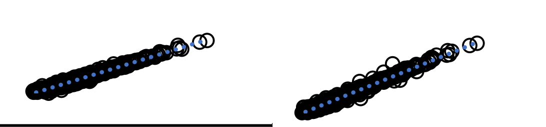	
25ps_BEC	0.95	0.99	0.99	0.99	0.99	0.99	1.00	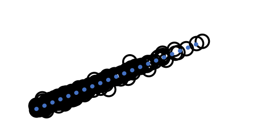
26ps_BEC	0.96	0.99	0.99	0.98	0.99	0.99	0.99	1.00

Supplementary Figure S1D.

	19c_BEC	*20c_BEC*	*21c_BEC*	*22c_BEC*	*23c_BEC*	*24c_BEC*	*25c_BEC*	*26c_BEC*
19c_BEC	1.00	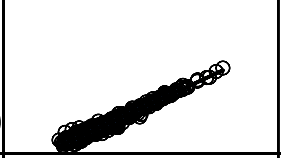	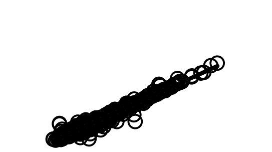	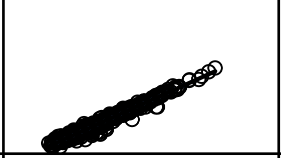	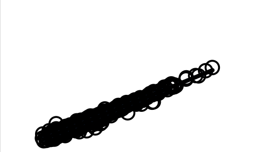	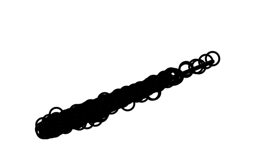	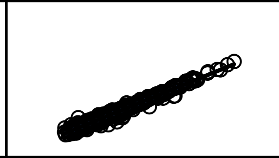	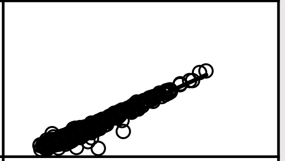
20c_BEC	0.99	1.00	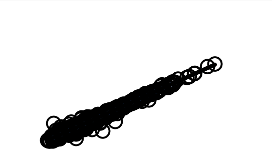	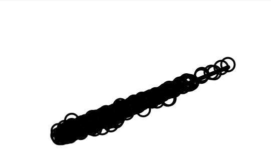	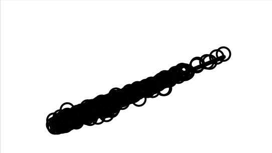	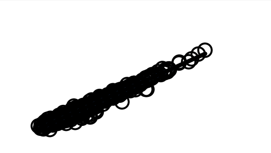	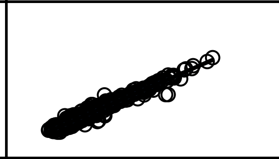	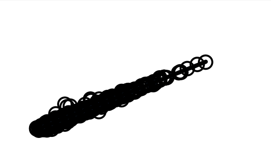
21c_BEC	0.98	0.99	1.00	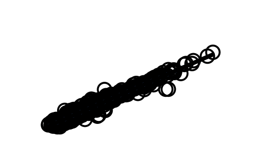	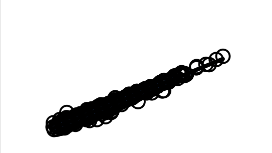	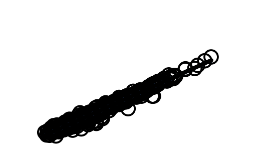	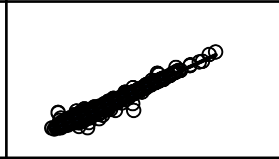	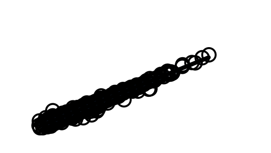
22c_BEC	0.98	0.98	0.98	1.00	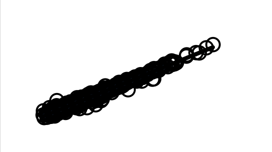	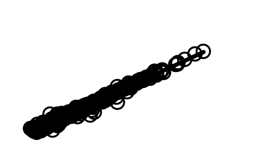	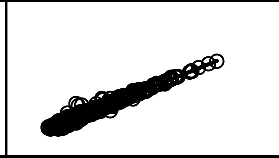	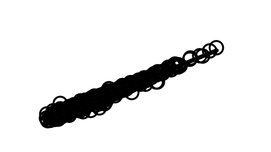
23c_BEC	0.99	0.99	0.98	0.99	1.00	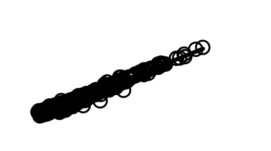	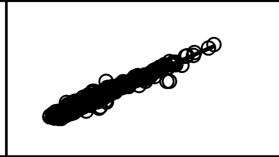	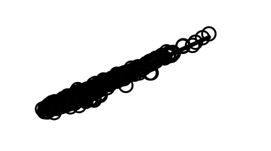
24c_BEC	0.98	0.98	0.98	0.99	0.99	1.00	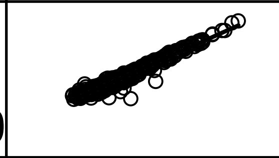	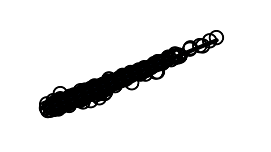
25c_BEC	0.98	0.98	0.97	0.99	0.98	0.98	1.00	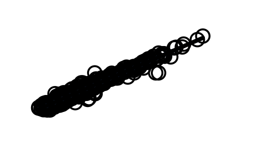
26c_BEC	0.98	0.98	0.98	0.98	0.98	0.99	0.98	1.00
